# Clinical characteristics of Chinese diabetic patients and their antibody responses to Omicron subvariants

**DOI:** 10.3389/fimmu.2026.1909061

**Published:** 2026-08-12

**Authors:** Lanxin Ma, Ruijing Bao, Wenjing Ruan, Rongrong Dai, Xinyu Liu, Nani Xu, Fang He, Jimin Sun, Haichang Yin, Hangjie Zhang

**Affiliations:** 1 School of Life Sciences and Agriculture and Forestry, Qiqihar University, Qiqihar, China; 2 Department of Prevention and Control of Infectious Disease, Zhejiang Provincial Center for Disease Control and Prevention, Hangzhou, China; 3 Zhejiang Institute of Integrated Traditional Chinese and Western Medicine, Zhejiang Hospital of Integrated Traditional Chinese and Western Medicine, Hangzhou, Zhejiang, China; 4 Department of Public Health and Preventive Medicine, Wuxi School of Medicine, Jiangnan University, Wuxi, China; 5 School of Public Health, Hangzhou Medical College, Hangzhou, China; 6 Department of Immunization Program, Xihu District Center for Disease Control and Prevention, Hangzhou, China; 7 Department of Clinical Laboratory, Zhejiang Provincial People’s Hospital, Hangzhou, Zhejiang, China; 8 Zhejiang Key Lab of Vaccines, Department of Prevention and Control of Infectious Disease, Zhejiang Provincial Center for Disease Control and Prevention, Hangzhou, China

**Keywords:** clinical characteristics, COVID-19, diabetes mellitus, humoral immune response, Omicron variant, pseudovirus neutralization assay

## Abstract

**Background:**

The ongoing circulation of SARS-CoV-2 Omicron sublineages continues to threaten vulnerable populations such as individuals with diabetes mellitus. Diabetes is well documented to impair host immune function; however, the correlation between clinical manifestations and cross-variant neutralizing immunity against evolving Omicron strains remains poorly characterized in diabetic patients during large-scale Omicron outbreaks.

**Methods:**

A cross-sectional study was conducted after the nationwide Omicron surge in China from April to June 2023. A total of 200 diabetic patients and 200 age-comparable healthy controls (HCs) were enrolled. We systematically compared their clinical symptoms, serum anti-RBD IgG levels, and neutralizing antibody (NAb) geometric mean titers (GMTs) against Omicron BA.4/5 and XBB.1.5 using a pseudovirus neutralization assay.

**Results:**

Clinically, diabetic patients exhibited a significantly lower prevalence of high-grade fever (≥38 °C, 11.8% vs. 46.1%, P<0.001) and myalgia (1.5% vs. 37.0%, P<0.001) compared with HCs. Immunologically, anti-RBD IgG concentrations were markedly reduced in the diabetic group (P<0.01). While NAb titers against BA.4/5 were comparable between the two cohorts (P>0.05), diabetic patients had drastically diminished neutralizing activity against the immune-evasive XBB.1.5 variant (GMT: 18.9 vs. 59.6 in HCs, P<0.001). Diabetic individuals also showed a far greater fold reduction in cross-variant neutralizing capacity from BA.4/5 to XBB.1.5 than HCs (P<0.001). Gender stratification revealed higher BA.4/5-specific NAb titers in female participants across both groups. Age was negatively associated with BA.4/5-specific NAb titers in both diabetic patients and healthy controls, whereas XBB.1.5 neutralization was uniformly low irrespective of age, which may reflect an age-independent susceptibility of diabetic patients to immune-evasive variants.

**Conclusions:**

Diabetic patients showed a distinct self-reported symptom profile, characterized by lower reported frequencies of high-grade fever and myalgia, but also exhibited reduced cross-variant neutralizing activity, particularly against the immune-evasive XBB.1.5 sublineage. The reduced XBB.1.5 neutralizing activity observed in diabetic patients highlights the need for targeted immunological monitoring and risk management during waves of immune-evasive SARS-CoV-2 variants.

## Introduction

Since the identification of severe acute respiratory syndrome coronavirus 2 (SARS-CoV-2) in 2019, successive epidemic waves driven by newly emerging variants have shaped the global trajectory of the COVID-19 pandemic. The accumulation of mutations within the spike protein has enabled these viruses to evade neutralizing antibodies generated through vaccination or previous infection (1–3), thereby facilitating their efficient spread even in populations with substantial prior immunity. Although Omicron infections are generally milder than those caused by earlier variants (4, 5), the virus retains strong replication capacity in the upper airway and demonstrates enhanced ACE2 binding affinity and pronounced immune-escape characteristics (6, 7). In China, the nationwide surge that followed the relaxation of strict containment measures at the end of 2022 led to rapid population-wide SARS-CoV-2 exposure across the country (8, 9).

Individuals with diabetic patients have long been recognized as a vulnerable group to severe outcomes from respiratory viral infections. Chronic hyperglycemia can compromise several components of innate and adaptive immunity, including neutrophil recruitment, phagocytic activity, and pathogen clearance (10, 11). During the early phases of the COVID-19 pandemic, diabetes consistently appeared as a key predictor of severe disease and mortality (12). The underlying mechanisms are thought to involve persistent low-grade inflammation, endothelial dysfunction, and dysregulated immune activation linked to hyperglycemia (5, 13). Conversely, SARS-CoV-2 infection itself may destabilize glucose metabolism, potentially precipitating new-onset diabetes or worsening pre-existing disease, creating a cycle that complicates management (14). As the SARS-CoV-2 Omicron variant continues to evolve, understanding the clinical and immunological responses to Omicron infection in individuals with diabetes remains critically important.

Despite increasing interest, data describing the clinical presentation and post-infection humoral immunity of diabetic individuals during the Omicron period remain scarce. Much of the available research has centered on vaccine-induced responses or focused on older adult populations (15, 16), leaving natural infection in diabetic populations relatively understudied. Given that individuals with diabetes may mount weaker immune responses or experience rapid antibody waning (15, 17), characterization of their antibody profiles against Omicron and its highly immune-evasive descendants is particularly important (1, 2). More recently, Omicron descendants such as JN.1 have further illustrated the continuous antigenic diversification of SARS-CoV-2, with accumulating spike mutations contributing to enhanced immune escape and renewed concerns regarding population-level susceptibility. These developments underscore the importance of monitoring variant-specific humoral immunity in high-risk populations, including individuals with diabetes (18, 19).

In this study, we compared infection-related symptoms and antibody responses to the Omicron BA.4/5 and XBB.1.5 sublineages between adults with diabetes mellitus and healthy controls during the major Omicron wave in China in early 2023. Our findings are intended to fill existing knowledge gaps and provide evidence to guide the development of tailored prevention strategies for populations at elevated risk of Omicron infection.

## Materials and methods

### Study design and participants

**Figure 1:**
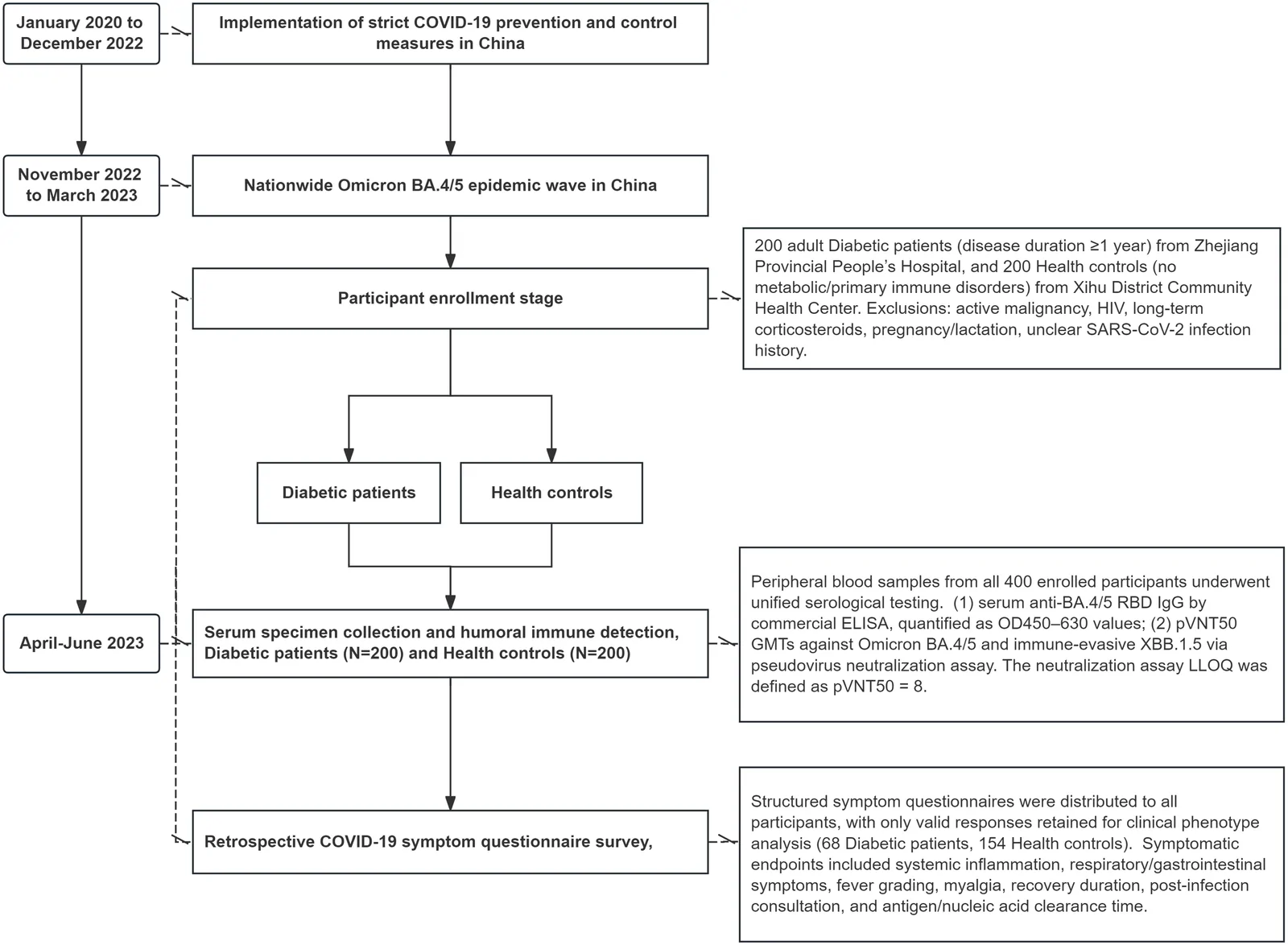
Study design. Flowchart showing the nationwide Omicron wave, the enrollment of diabetic patients and healthy controls, blood collection, antibody assessment, and symptom questionnaire survey. Blood samples were collected from all enrolled participants for anti-RBD IgG measurement and pseudovirus neutralization assays against BA.4/5 and XBB.1.5.

This cross-sectional study was performed in Zhejiang Province, China, spanning the period from April to June 2023, with the overall research timeline shown in **Figure 1**. Two groups of adult participants were enrolled: 200 patients with a clinically confirmed diagnosis of diabetes mellitus were recruited from Zhejiang Provincial People’s Hospital, and 200 community-dwelling healthy adults presenting for routine physical examinations at Xihu Community Health Center were selected via convenience sampling to form the control group. Eligible participants were 18 years of age or older; for the diabetes mellitus group, confirmed diagnosis of ≥1 year was required. Controls were screened to exclude diabetes and other immune-compromising conditions (20, 21).

Participants were excluded if their history of SARS-CoV-2 infection could not be reliably determined or if they had active malignancy, HIV infection, marked immunodeficiency, or had received immunosuppressive treatment, including prolonged corticosteroid use, within the previous six months. Pregnant or breastfeeding women were also excluded. Written informed consent was obtained from all participants before enrollment, and the study protocol received approval from the institutional ethics committee.

To classify participants as infected or uninfected with SARS-CoV-2 during the nationwide Omicron BA.4/5 epidemic wave in China, serological evidence was adopted for definitive classification rather than questionnaire-based self-reporting.

### Immunological and antibody assessment

Neutralizing activity against the SARS-CoV-2 Omicron BA.4/5 and XBB.1.5 sublineages was assessed using a pseudovirus-based neutralization assay. Spike proteins from each Omicron sublineage were codon-optimized and cloned into the pCAGGS vector. HEK-293T cells were transfected with 30 μg of each construct, and after 24 hours, cells were exposed to VSV-ΔG-GFP pseudovirus. The inoculum was removed two hours later and replaced with complete DMEM supplemented with an anti-VSV-G antibody (I1-Hybridoma, ATCC CRL-2700) to eliminate residual input virus. Supernatants harvested 30 hours after infection were filtered through a 0.45-μm membrane (Millipore SLHP033RB), aliquoted, and stored at −80 °C until further use.

For neutralization testing, serum samples were first diluted 1:8 and then serially 2-fold diluted in 96-well plates. After incubation with pseudovirus at 37 °C for one hour, the mixtures were transferred onto Vero cell monolayers. Fluorescent transduction units were quantified 20 hours later using an EVOS M7000 imaging system, and pVNT50 values were calculated via nonlinear regression analysis using GraphPad Prism. Geometric mean titers were derived from the distribution of pVNT50 values across participants. The Lower Limit of Quantitation (LLOQ) of the pseudovirus neutralization assay was defined as a pVNT50 of 8, corresponding to the initial serum dilution of 1:8. Values below the LLOQ were assigned a value of 4 before GMT calculation and log2 transformation, whereas samples without valid fitted pVNT50 values after retesting were treated as missing values. Every sample was measured in duplicate technical replicates; additional triplicate testing was performed for replicates with high deviation to secure reliable titer outcomes.

Serum anti-RBD IgG levels were determined with a commercial ELISA targeting the Omicron BA.4/5 RBD (Nanjing Vazyme Biotech Co., Ltd. Nanjing, China). Plates pre-coated with recombinant RBD antigen were incubated with serially diluted serum samples and the corresponding positive and negative controls at 37 °C for one hour. After washing, plates were incubated with HRP-conjugated secondary antibody for 30 minutes, followed by TMB substrate development for 15 minutes. Reactions were stopped, and optical density was measured at 450/630 nm. RBD-specific IgG responses were expressed as OD_450-630_. In accordance with the assay kit manufacturer’s recommendations and established prior literature, an OD_450-630_ value of ≥ 0.165 was set as the seropositivity cutoff (9, 22).

### Statistical analysis

We compiled demographic characteristics and clinical outcomes for all participants in both study groups; categorical data, such as gender, age distribution, and vaccination history, were reported as absolute numbers and percentages. Before conducting statistical tests, we assessed continuous variable distributions: anti-RBD IgG levels, which did not follow a normal distribution, were summarized as medians with interquartile ranges (IQR) and compared using the nonparametric Mann-Whitney U test, while neutralizing antibody activity data were presented as GMTs with 95% confidence intervals (CI). To meet the assumptions for parametric analysis, pVNT50 titers were log2-transformed prior to evaluation via Student’s t-tests or one-way analysis of variance (ANOVA). For paired comparisons of neutralizing activity between different Omicron subvariants (BA.4/5 vs. XBB.1.5) within the same individual, we used the Wilcoxon matched-pairs signed-rank test. Cross-variant neutralization change was calculated for each participant as the BA.4/5-to-XBB.1.5 pVNT50 ratio, using the formula BA.4/5 pVNT50 divided by XBB.1.5 pVNT50 after below-LLOQ handling. Between-group differences in this ratio were assessed using the two-tailed Mann-Whitney U test. All statistical tests were two-sided, and a P value < 0.05 was considered statistically significant, and all analyses were performed using SAS 9.4 (SAS Institute Inc., Cary NC, USA) and Prism software version 9.0.2 (GraphPad Software, Inc., San Diego CA, USA).

## Results

### Demographic characteristics of the study population

The present study enrolled 400 subjects in total, split equally into 200 patients diagnosed with diabetes mellitus and 200 healthy individuals serving as control subjects (**Table 1**). Sex distribution differed significantly between the two groups, with a higher proportion of male participants in the diabetic group than in the healthy control group (65.5% vs. 51.5%, P = 0.005). The median age was 61 years (IQR: 51.5–69) in diabetic patients and 67 years (IQR: 47.5–73) in healthy controls, and no statistically significant between-group difference in age was observed (P > 0.05).

**Table 1:** Demographic characteristics of participants.

Variable	Diabetic patients (N = 200)	Healthy controls (N = 200)	P
Sex			
Male	131 (65.5%)	103 (51.5%)	0.005
Female	69 (34.5%)	97 (48.5%)	
Age, years			
18-59	95 (47.5%)	68 (34.0%)	0.006
≥60	105 (52.5%)	132 (66.0%)	
Median (P25,P75)	61 (51.5,69)	67 (47.5,73)	
BMI (kg/m^2^)^a^			
<25	44 (22.0%)	142 (71.0%)	–
≥25	24 (12.0%)	57 (28.5%)	
unknown	132 (66.0%)	1 (0.5%)	
Vaccine(s) administered^a^			
Yes	178 (89.0%)	198 (99.0%)	–
No	22 (11.0%)	2 (1.0%)	
COVID-19 infection^b^			
Yes	190 (95.0%)	184 (92.0%)	0.224
No	10 (5.0%)	16 (8.0%)	

Data are presented as n (%) or median (IQR). Between-group comparisons were performed using Pearson’s chi-square test or two-sided Fisher’s exact test for categorical variables Statistical significance was defined as P < 0.05.

^a^ Valid between-group comparisons of BMI (dichotomized as <25 kg/m² vs. ≥25 kg/m²) were not feasible owing to substantial and differential missing data. For subgroups with very small sample sizes, we have removed inferential statistical comparisons (P values).

^b^ Participants’ SARS-CoV-2 infection status during China’s nationwide Omicron BA.4/5 wave was ascertained via serological assays instead of questionnaire-based reporting.

BMI data were missing in 132 diabetic patients (66.0%) and 1 healthy control (0.5%); therefore, BMI was presented descriptively and was not formally compared between groups. Vaccination status differed significantly between groups, with a lower vaccination rate in diabetic patients than in healthy controls (89.0% vs. 99.0%, P < 0.001). The proportion of participants with SARS-CoV-2 infection was comparable between diabetic patients and healthy controls (95.0% vs. 92.0%, P > 0.05).

### Clinical characteristics of COVID-19 infection and associated factors

We further evaluated clinical manifestations and recovery trajectories in a predefined subset of participants, consisting of 68 diabetic patients and 154 healthy controls (**Table 2**). No statistically meaningful between-group differences emerged for the prevalence of systemic symptoms (79.4% vs 84.9%, P = 0.243), respiratory complaints (60.3% vs 49.4%, P = 0.147), or gastrointestinal symptoms (0.0% vs 5.2%, P = 0.110). However, high-grade fever, defined as body temperature ≥ 38 °C, was reported less frequently by diabetic patients than by healthy controls (11.8% vs. 46.1%, P < 0.001). Myalgia was also less frequently reported by diabetic patients than by healthy controls (1.5% vs. 37.0%, P < 0.001). Symptom recovery within one week was reported by 88.2% of diabetic patients and 78.6% of healthy controls, but the difference did not reach statistical significance (P = 0.087). No obvious intergroup divergence was detected in healthcare consultation rates after infection, with 11.8% of diabetic patients and 20.1% of control subjects sought medical consultation after SARS-CoV-2 infection (P = 0.131). Similarly, the interval needed for SARS-CoV-2 antigen or PCR testing to convert from positive to negative showed no intergroup disparity, and almost all enrolled participants in both groups attained virological clearance within three weeks (P = 1.000).

**Table 2:** Clinical symptoms of participants.

Variable	Diabetic patients (N = 68)	Healthy controls (N = 154)	P
General symptom			
Yes	54 (79.4%)	132 (84.9%)	0.243
No	14 (20.6%)	22 (15.1%)	
Respiratory symptom			
Yes	41 (60.3%)	76 (49.4%)	0.147
No	27 (39.7%)	78 (50.6%)	
Digestive tract symptom			
Yes	0 (0.0%)	8 (5.2%)	0.110
No	68 (100.0%)	146 (94.8%)	
Fever			
<38 °C	60 (88.2%)	83 (53.9%)	<0.001
≥38 °C	8 (11.8%)	71 (46.1%)	
Muscular soreness			
Yes	1 (1.5%)	57 (37.0%)	<0.001
No	67 (98.5%)	97 (63.0%)	
Symptom recovery time			
<1 weeks	60 (88.2%)	121 (78.6%)	0.087
≥1 weeks	8 (11.8%)	33 (21.4%)	
Medical visit after COVID-19 infection			
Yes	8 (11.8%)	31 (20.1%)	0.131
No	60 (88.2%)	123 (79.9%)	
Time of SARS-CoV-2 antigen or PCR turns to negative^a^			
<3 weeks	68 (100%)	153 (99.4%)	–
≥3 weeks	0 (0.0%)	1 (0.6%)	

Data are presented as n (%). Analyses were restricted to participants who completed the symptom questionnaire, including 68 diabetic patients and 154 healthy controls. Between-group comparisons were performed using Pearson’s chi-square test or two-sided Fisher’s exact test, as appropriate. Fever was defined as self-reported body temperature ≥38°C. Statistical significance was defined as P < 0.05.

^a^ For subgroups with very small sample sizes, we have removed inferential statistical comparisons (P values) and retained only descriptive data.

We analyzed the correlations linking demographic parameters to COVID-19 symptomatic profiles in patients with diabetes (**Table 3**; **Supplementary Table 1**). Sex exerted no statistically significant influence on the incidence of any recorded symptom: constitutional symptoms occurred in 81.4% of male participants versus 76.0% of females (P = 0.596), respiratory symptoms in 55.8% versus 68.0% (P = 0.322), high-grade fever in 11.6% versus 12.0% (P = 1), and myalgia in 2.3% versus 0.0% (P = 1.000). When stratified by age, younger diabetic patients aged 18–59 years reported higher frequencies of respiratory symptoms and high-grade fever than those aged ≥60 years. Respiratory symptoms were reported by 25 of 31 patients aged 18–59 years and by 16 of 37 patients aged ≥60 years (80.6% vs. 43.2%, P = 0.012), while high-grade fever was reported by 7 of 31 and 1 of 37 patients, respectively (22.6% vs. 2.7%, P = 0.020). Further grouping by BMI and COVID-19 vaccination status revealed that neither metric was associated with the development of the four above-mentioned symptom types, as all corresponding comparisons returned P values above 0.05.

**Table 3:** Factors associated with COVID-19 symptoms in diabetic patients.

Variable	COVID-19 infection		General symptom		Respiratory symptom		Fever (≥38°C)^a^		Muscular soreness^a^	
	N (%)	P	N (%)	P	N (%)	P	N (%)	P	N (%)	P
Sex										
Male	125/131 (95.4%)	0.740	35/43 (81.4%)	0.596	24/43 (55.8%)	0.322	5/43 (11.6%)	–	1/43 (2.3%)	–
Female	65/69 (94.2%)		19/25 (76.0%)		17/25 (68.0%)		3/25 (12.0%)		0/25 (0.0%)	
Age, years										
18-59	92/95 (96.8%)	0.337	21/31 (67.7%)	0.589	24/31 (77.4%)	0.012	7/31 (22.6%)	–	0/31 (0.0%)	–
≥60	98/105 (93.3%)		28/37 (75.7%)		17/37 (45.9%)		1/37 (2.7%)		1/37 (2.7%)	
BMI (kg/m^2^)										
<25	44/44 (100.0%)	1	35/44 (79.5%)	0.971	26/44 (59.1%)	0.784	5/44 (11.4%)	–	0/44 (0.0%)	–
≥25	24/24 (100.0%)		19/24 (79.2%)		15/24 (62.5%)		3/24 (12.5%)		1/24 (4.2%)	
COVID-19 Vaccine (s) administered										
Yes	170/178 (95.5%)	0.303	51/65 (78.5%)	1	39/65 (60.0%)	1	8/65 (12.3%)	–	1/65 (1.5%)	–
No	20/22 (90.9%)		3/3 (100.0%)		2/3 (66.7%)		0/3 (0.0%)		0/3 (0.0%)	

Data are presented as n/N (%) or n (%), as appropriate. The analysis of SARS-CoV-2 infection included all enrolled participants, whereas analyses of clinical symptoms were restricted to participants who completed the symptom questionnaire. Percentages for symptom-related variables were calculated using the number of respondents in each subgroup as the denominator. Associations were evaluated using Pearson’s chi-square test or two-sided Fisher’s exact test, as appropriate. Fisher’s exact test was used for fever and muscular soreness because of small event counts in several subgroups. All statistical tests were two-sided, and P < 0.05 was considered statistically significant.

^a^ For subgroups with very small sample sizes, we have removed inferential statistical comparisons (P values) and retained only descriptive data.

### Antibody responses to SARS-CoV-2 Omicron variants

**Figure 2:**
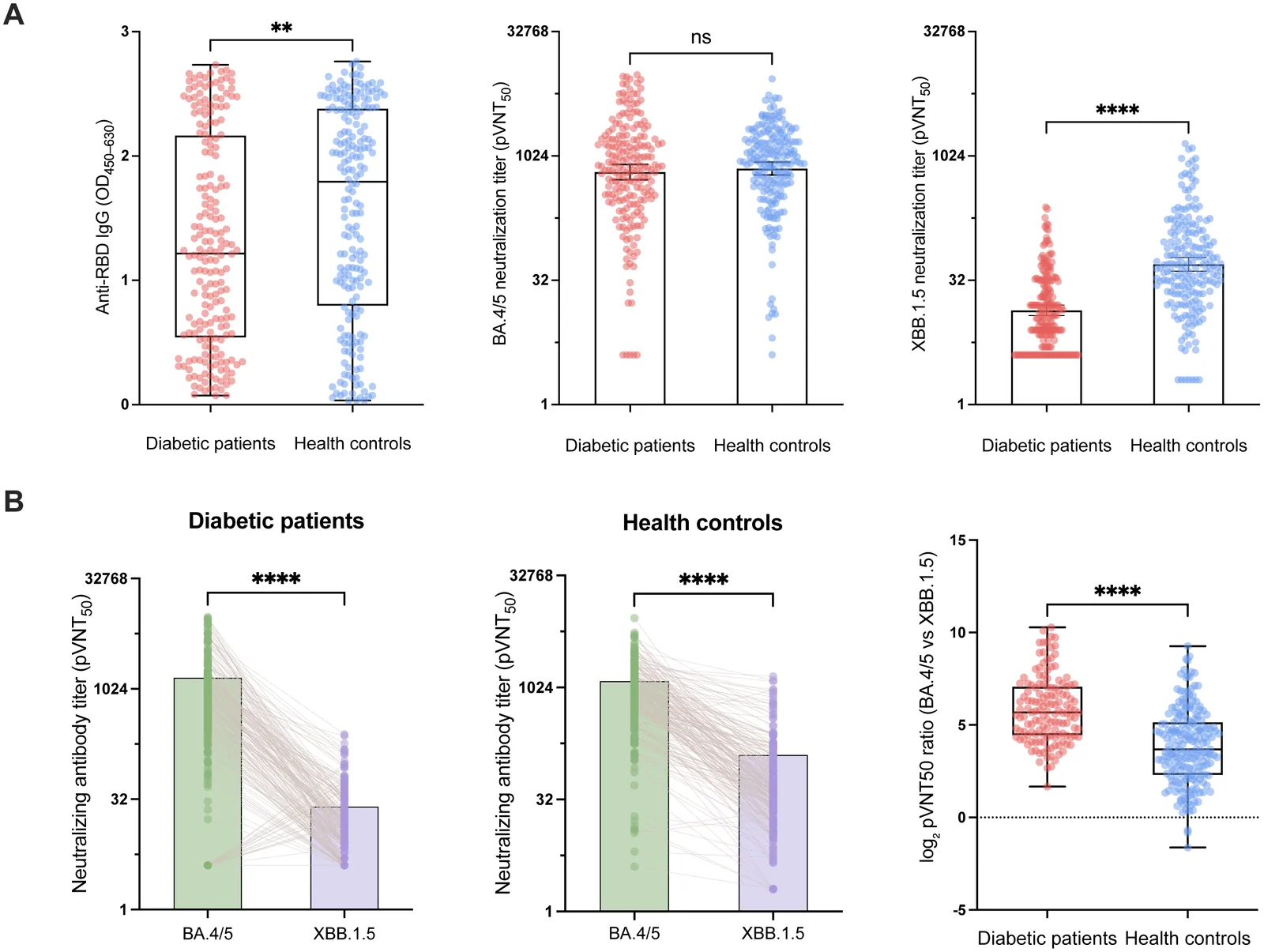
Humoral immune responses to SARS-CoV-2 Omicron variants. **(A)** Comparison of anti-RBD IgG levels and neutralizing antibody (NAb) titers against BA.4/5 and XBB.1.5 between diabetic patients and healthy controls. **(B)** Paired comparison of BA.4/5- and XBB.1.5-specific NAb titers within each group, and comparison of the within-individual log_2_ pVNT50 ratio between groups. The log_2_ pVNT50 ratio was calculated as log_2_ (BA.4/5 pVNT50/XBB.1.5 pVNT50). Anti-RBD IgG levels are expressed as OD_450–630_ values. For NAb titer plots, pVNT50 values are shown on a logarithmic scale. Boxes indicate medians and interquartile ranges, whiskers indicate ranges, and dots represent individual participants. Between-group comparisons were performed using the Mann–Whitney U test, and paired comparisons were performed using the Wilcoxon matched-pairs signed-rank test. ns, not significant; **P < 0.01; ****P < 0.001.

To delineate the antibody response status of all study participants, we compared serum anti-RBD IgG levels and NAb titers between diabetic patients and healthy controls (**Figure 2A**). Diabetic patients exhibited significantly reduced anti-RBD IgG levels relative to healthy controls, with a median value of 1.3 (IQR: 0.6–2.2) versus 1.8 (IQR: 0.8–2.4) in the control group (P < 0.01). In terms of variant-specific neutralizing capacity, no significant intergroup difference was observed in NAb titers against the Omicron BA.4/5 sublineage (P > 0.05). Diabetic patients presented a BA.4/5-specific GMT of 861.2 (95% CI: 711.9–1042.0), which was comparable to the GMT of 719.8 (95% CI: 601.4–861.4) in healthy individuals. In contrast, diabetic patients showed substantially impaired neutralizing activity against the highly immune-evasive XBB.1.5 variant. The XBB.1.5-specific GMT was only 18.9 (95% CI: 16.3–21.8) in diabetic patients, which was significantly lower than the corresponding GMT of 59.6(95% CI: 49.67–71.47) in healthy controls (P < 0.001).

Neutralizing titers targeting BA.4/5 were significantly higher than those for XBB.1.5 within both study groups (P < 0.001) (**Figure 2B**). Among diabetic patients, the GMT for BA.4/5 reached 861.2 (95% CI, 711.9–1042.0), whereas this value plummeted to 18.9 (95% CI, 16.3–21.8) for XBB.1.5. Importantly, diabetic subjects exhibited a substantially greater fold decline in cross-variant neutralizing capacity relative to healthy controls (P < 0.001), pointing to increased susceptibility to SARS-CoV-2 variants with prominent antigenic drift.

We also conducted an analysis of factors affecting diabetes status. After including gender, age, and vaccination status, the results indicate that neutralizing antibody titers against BA.4/5 and XBB.1.5 in patients with diabetes were significantly lower compared to the healthy population (**Supplementary Table 2**).

### Factors associated with antibody levels

**Figure 3:**
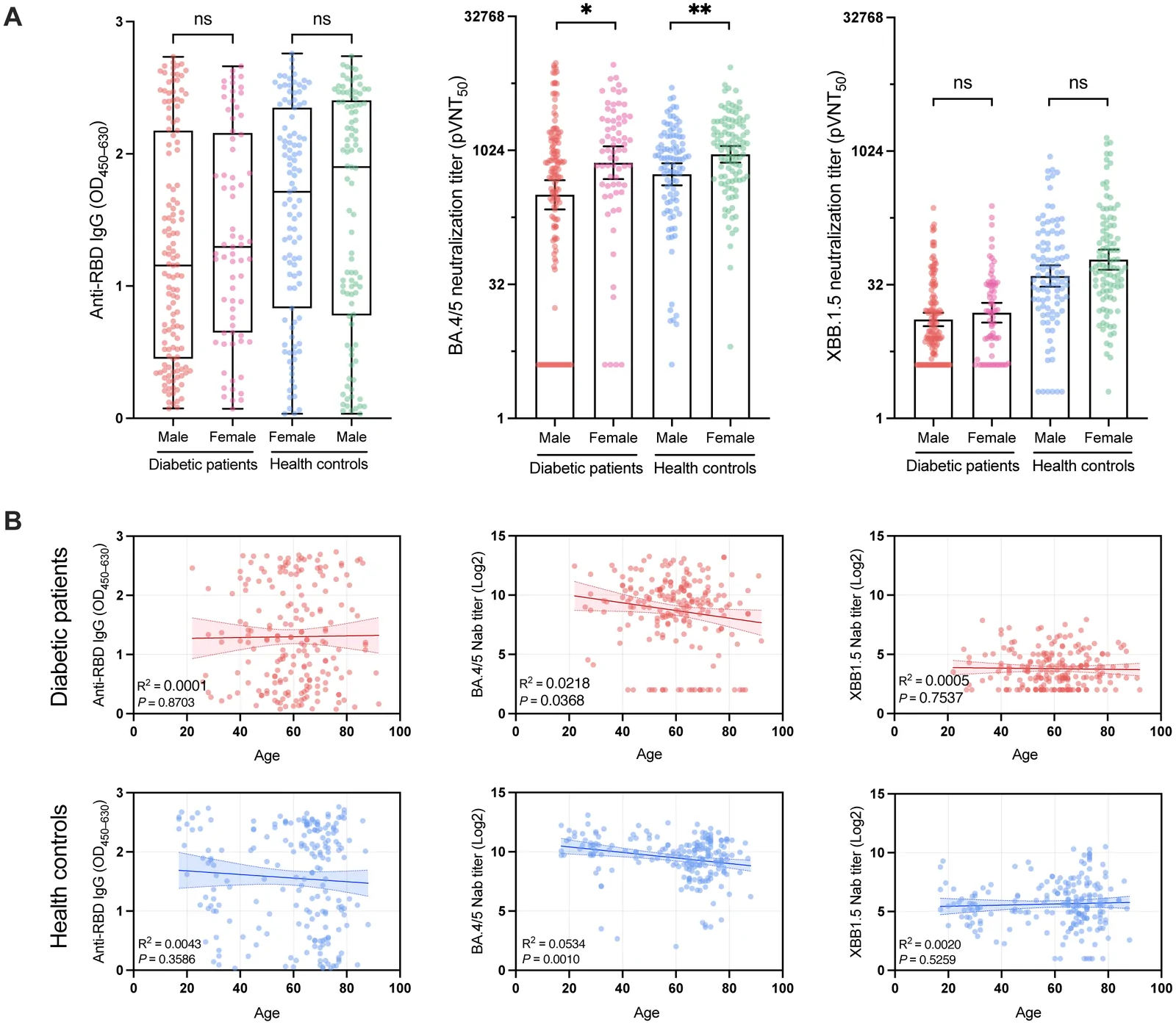
Associations of sex and age with SARS-CoV-2 antibody levels. **(A)** Sex-stratified comparison of anti-RBD IgG levels and neutralizing antibody titers against BA.4/5 and XBB.1.5 in diabetic patients and healthy controls. **(B)** Unadjusted age-related correlation analyses of antibody responses. Box-and-whisker plots show the median and interquartile range, with whiskers extending to the range of data. Scatter plots display individual data points, with solid lines representing linear regression fits and shaded areas indicating the 95% confidence intervals. P values are shown in each panel. Between-group comparisons were performed using the Mann–Whitney U test. ns, not significant; *P < 0.05, **P<0.01.

We analyzed independent factors correlated with the magnitude of antibody responses against SARS-CoV-2 among enrolled participants (**Figure 3**; **Table 4**; **Supplementary Table 3**). Sex was associated with BA.4/5 NAb titers, with female participants exhibiting notably higher titers than male counterparts (744.6 vs. 324.5, P = 0.003) in the diabetic group, whereas sex exerted no significant effect on anti-RBD IgG levels (P = 0.377) or XBB.1.5 NAb titers (P = 0.224). Unadjusted linear regression analyses were further performed to evaluate the association between age and antibody responses (**Figure 3B**). In both diabetic patients and healthy controls, age was significantly negatively correlated with BA.4/5-specific NAb titers, indicating lower BA.4/5 neutralizing activity with increasing age in both groups (diabetic patients: P = 0.0368; healthy controls: P = 0.0010). In contrast, age was not significantly associated with anti-RBD IgG levels or XBB.1.5-specific NAb titers in either group. Additionally, COVID-19 vaccination status showed minimal influence on the antibody levels, with no significant differences in anti-RBD IgG and BA.4/5 NAb titers detected between vaccinated and unvaccinated individuals (all P > 0.05), whereas unvaccinated participants presented elevated XBB.1.5 neutralizing GMT relative to vaccinated counterparts (P = 0.047).

**Table 4:** Factors associated with SARS-CoV-2 antibody levels in diabetic patients.

Variable	Anti-RBD IgG		BA.4/5		XBB.1.5	
	Titer	P	GMT	P	GMT	P
Sex						
Male	1.2 (0.5,2.2)	0.377	324.5 (222.2,474.0)	0.003	13.0 (10.9,15.5)	0.224
Female	1.3 (0.6,2.2)		744.6 (487.1,1138)		15.5 (12.0,20.0)	
Age, years						
18-59	1.3 (0.5,2.2)	0.958	541.2 (363.0,806.8)	0.168	13.9 (11.3,16.8)	0.973
≥60	1.2 (0.6,2.2)		352.6 (230.9,538.4)		13.7 (11.3,17.2)	
COVID-19 Vaccines administered						
Yes	1.2 (0.6,2.2)	0.490	411.9 (299.5,566.4)	0.417	13.5 (11.6,15.7)	0.294
No	1.0 (0.5,2.0)		637.7 (336.9,1206.8)		16.9 (10.4,27.6)	

Data are presented as median (IQR). Statistical analyses were performed using the non-parametric Mann-Whitney U test. Data for pseudovirus neutralization titers (pVNT_50_) against Omicron subvariants (BA.4/5 and XBB.1.5) and are presented as GMTs with 95% CI. Statistical significance for pVNT50 was determined using unpaired t-tests or one-way ANOVA following log2 transformation. A P value < 0.05 was considered statistically significant.

Multivariable linear regression models were independently established for individuals with diabetes and healthy controls to explore the correlations between participant baseline characteristics and antibody responses (**Table 5**). Age, sex, and COVID-19 vaccination status were incorporated as explanatory variables in the regression analysis. In both study groups, advanced age was significantly correlated with reduced BA.4/5-specific NAb titers, and sex also exhibited a robust association with BA.4/5-specific NAb levels. Notably, these correlations remained statistically significant in both diabetic patients (age: P = 0.0424; sex: P = 0.0056) and healthy controls (age: P = 0.0015; sex: P = 0.0068). The negative coefficient for the sex variable indicated that males harbored lower BA.4/5-specific NAb titers relative to females. By contrast, no stable or significant correlations were detected between age, sex, or vaccination status and anti-RBD IgG levels or XBB.1.5-specific NAb titers across the two groups.

**Table 5:** Multivariable regression analysis of factors associated with antibody responses in the diabetic patients and healthy controls.

Antibody response	Variable	B	95%CI	P value
Diabetic patients				
Anti-RBD IgG	Age, years	-0.0003	-0.0089 — 0.0083	0.9447
	Sex	-0.1147	-0.3636 — 0.1343	0.3647
	Vaccine(s) administered	0.1423	-0.2357 — 0.5202	0.4588
BA.4/5	Age, years	-0.0310	-0.0609 — -0.0011	0.0424
	Sex	-1.2290	-2.0950 — -0.3629	0.0056
	Vaccine(s) administered	-0.4961	-1.8110 — 0.8186	0.4577
XBB.1.5	Age, years	-0.0027	-0.0178 — 0.0125	0.7296
	Sex	-0.2468	-0.6860 —0.1924	0.2691
	Vaccine(s) administered	-0.3067	-0.9735 — 0.3600	0.3654
Healthy controls				
Anti-RBD IgG	Age, years	-0.0031	-0.0097 — 0.0035	0.3516
	Sex	-0.0094	-0.2519 — 0.2332	0.9392
	Vaccine(s) administered	0.2340	-0.9854 — 1.4530	0.7056
BA.4/5	Age, years	-0.0222	-0.0358 — -0.0086	0.0015
	Sex	-0.6952	-1.1960 — -0.1942	0.0068
	Vaccine(s) administered	-0.6497	-3.1690 — 1.8690	0.6115
XBB.1.5	Age, years	0.0071	-0.0074 — 0.0215	0.3374
	Sex	-0.5255	-1.0580 — 0.0068	0.0530
	Vaccine(s) administered	-2.4900	-5.1650 — 0.1863	0.0680

Data are presented as regression coefficients (B), 95% confidence intervals (95% CI), and P values. Multivariable linear regression analyses were performed separately in diabetic patients and healthy controls to evaluate associations between participant characteristics and antibody responses. Neutralizing antibody titers against BA.4/5 and XBB.1.5 were log_2_-transformed before analysis. Statistical significance was defined as P < 0.05.

## Discussion

The continuous evolution of SARS-CoV-2 Omicron sublineages with enhanced immune escape capability has posed persistent public health challenges worldwide, especially for immunocompromised and metabolically disordered populations (23). This large cross-sectional study characterized clinical manifestations and humoral immune responses to Omicron BA.4/5 and XBB.1.5 in Chinese diabetic patients and healthy controls during the 2022–2023 national Omicron wave, and revealed a discrepancy between selected self-reported symptom profiles and antiviral humoral immune responses in diabetic individuals.

In this study, diabetic patients reported lower frequencies of high-grade fever and myalgia during Omicron infection than healthy controls (24). Fever and myalgia are common systemic symptoms during SARS-CoV-2 infection and may be influenced by host inflammatory responses, symptom perception, reporting behavior, and clinical background. Previous studies have suggested that chronic hyperglycemia may impair innate immune responses, including neutrophil recruitment, phagocytic activity, and pro-inflammatory mediator production (25). These mechanisms may partly explain the lower reported frequencies of high-grade fever and myalgia observed in diabetic patients in our study. However, because glycemic control markers and immune cell functions were not measured, this explanation remains speculative and cannot be directly verified by our data. Therefore, lower reporting of selected symptoms should not be interpreted as indicating low infection risk or preserved antiviral protection (15, 23).

Humoral immunity is the primary line of defense against SARS-CoV-2 infection and reinfection. Pseudovirus neutralization assays, the gold standard for evaluating variant-specific neutralizing activity, showed that BA.4/5-specific NAb titers were comparable between diabetic patients and healthy controls. However, when challenged by the highly immune-evasive XBB.1.5 subvariant, diabetic patients exhibited a profound decline in neutralizing potency, with a significantly higher fold reduction in cross-variant neutralizing capacity than healthy individuals. This finding aligns with global reports that XBB lineage variants carry extensive spike protein mutations that disrupt the binding of existing neutralizing antibodies (15, 25). More importantly, our results further confirm that diabetes-related immune dysregulation is variant-dependent: such impairment is markedly amplified when facing highly mutated, immune-evasive SARS-CoV-2 strains. Chronic hyperglycemia not only disturbs innate immunity but also impairs B cell differentiation, antibody affinity maturation and memory B cell formation after viral exposure (15, 26), which directly weakens the breadth of cross-protective humoral immunity against drifted variants.

Stratified analysis by sex and age provided novel insights into population-based immune heterogeneity. In line with multiple immunological studies, female participants in both cohorts generated higher BA.4/5-specific NAb titers than males, a phenomenon generally explained by sex hormone regulation of immune cell function and stronger adaptive immune responses in females (27). By contrast, this sex-associated difference was less apparent for XBB.1.5-specific NAb titers. Compared with BA.4/5, XBB.1.5 showed markedly stronger immune-evasive properties and substantially lower neutralizing titers overall, particularly in diabetic patients. Under this condition, the sex-related difference observed for BA.4/5 became less distinguishable, suggesting that variant-specific antigenic escape may weaken the observable contribution of host sex-related immune differences to neutralizing antibody responses.

Age is a well-recognized factor affecting antiviral immunity in the general population (28). In both diabetic patients and healthy controls, older age was associated with lower BA.4/5-specific NAb titers, which is consistent with the influence of immunosenescence on antiviral humoral responses. In contrast, the age-related pattern was less clear for XBB.1.5-specific NAb titers. This may be because XBB.1.5 has stronger immune-evasive capacity than BA.4/5 and induces substantially lower neutralizing titers overall, making age-related differences less apparent.

Previous natural SARS-CoV-2 infection was identified as the strongest determinant of anti-RBD IgG and neutralizing antibody levels in our cohort, which is consistent with findings from multiple global cohort studies (29). Given the weakened cross-variant immunity in diabetic patients, regular updating of COVID-19 vaccines to match circulating dominant variants is essential to restore protective humoral immunity for this high-risk group (15, 30).

Several limitations should be acknowledged. First, although the study included diabetic patients and healthy controls of comparable age, the two groups were not strictly matched, and differences in sex and age distribution and vaccination status may have introduced residual confounding. Second, symptom-related analyses were based only on participants who completed the questionnaire, and the response rates differed between diabetic patients and healthy controls. Therefore, non-response bias, recall bias, and incomplete clinical information may have affected the interpretation of symptom-related findings. The anti-nucleocapsid antibody testing, formal PCR or rapid antigen test records were not available for retrospective verification in this study Third, several important diabetes-related clinical variables were unavailable, including BMI in a substantial proportion of diabetic patients, diabetes type, disease duration, HbA1c, fasting blood glucose, medication use, complications, and comorbidities. Although sensitivity analyses were performed among participants with available BMI data, BMI- and diabetes-severity-related findings should still be interpreted cautiously. Finally, this was an observational cross-sectional study that assessed humoral immune responses at a single time point. Causal relationships could not be inferred, and cellular immune responses, antibody durability, and longitudinal immune dynamics were not evaluated. Future prospective studies with better-matched cohorts, complete metabolic phenotyping, cellular immune assays, and longitudinal follow-up are needed to validate these findings.

In conclusion, diabetic patients presented a distinct self-reported symptom profile, characterized by lower frequencies of high-grade fever and myalgia, alongside reduced cross-variant neutralizing activity, particularly against the immune-evasive XBB.1.5 sublineage. Importantly, milder symptoms did not reflect preserved antiviral protection. These results underscore the need for targeted immunological monitoring and risk management for diabetic populations during future waves of immune-evasive SARS-CoV-2 variants.

## Data Availability

The original contributions presented in the study are included in the article/**Supplementary Material**. Further inquiries can be directed to the corresponding authors.

## References

[1] Li Y Qiao S Dong L Zhang R Li R Qin S . Antibody response assessment of immediate breakthrough infections after zero-COVID policy adjustment in China. Lancet Regional Health - Western Pacific. (2023) 40:100945. doi: 10.1016/j.lanwpc.2023.100945 38033432 PMC10684796

[2] Wang Q Iketani S Li Z Guo Y Yeh AY Liu M . Antigenic characterization of the SARS-CoV-2 Omicron subvariant BA.2.75. Cell Host Microbe. (2022) 30:1512–1517.e4. doi: 10.1016/j.chom.2022.09.002 36108630 PMC9444898

[3] Søraas A Grødeland G Granerud BK Ueland T Lind A Fevang B . Breakthrough infections with the omicron and delta variants of SARS-CoV-2 result in similar re-activation of vaccine-induced immunity. Front Immunol. (2022) 13:964525. doi: 10.3389/fimmu.2022.964525 36159859 PMC9493489

[4] Huang S Gao Z Wang S . China’s COVID-19 reopening measures—warriors and weapons. Lancet. (2023) 401:643–4. doi: 10.1016/S0140-6736(23)00213-1 PMC9949835 36841613

[5] Wolter N Jassat W Walaza S Welch R Moultrie H Groome M . Early assessment of the clinical severity of the SARS-CoV-2 omicron variant in South Africa: a data linkage study. Lancet. (2022) 399:437–46. doi: 10.1016/s0140-6736(22)00017-4 35065011 PMC8769664

[6] Hong Q Han W Li J Xu S Wang Y Xu C . Molecular basis of receptor binding and antibody neutralization of Omicron. Nature. (2022) 604:546–52. doi: 10.1038/s41586-022-04581-9 35228716

[7] Srivastava A Rockman-Greenberg C Sareen N Lionetti V Dhingra S . An insight into the mechanisms of COVID-19, SARS-CoV2 infection severity concerning β-cell survival and cardiovascular conditions in diabetic patients. Mol Cell Biochem. (2022) 477:1681–95. doi: 10.1007/s11010-022-04396-2 35235124 PMC8889522

[8] Chen Y Lin Y Lu H Wu X Pan Y Xia A . Real-world effectiveness of molnupiravir, azvudine and paxlovid against mortality and viral clearance among hospitalized patients with COVID-19 infection during the omicron wave in China: A retrospective cohort study. Diagn Microbiol Infect Dis. (2024) 109:116353. doi: 10.1016/j.diagmicrobio.2024.116353 38776665

[9] Zhang H Xu N Xu Y Qin P Dai R Xu B . Safety and immunogenicity of Ad5-nCoV immunization after three-dose priming with inactivated SARS-CoV-2 vaccine in Chinese adults. Nat Commun. (2023) 14:4757. doi: 10.1038/s41467-023-40489-2 37553338 PMC10409730

[10] Chen Y Song W Li C Wang J Liu F Ye Z . COVID-19 mRNA vaccine protects against SARS-CoV-2 Omicron BA.1 infection in diet-induced obese mice through boosting host innate antiviral responses. eBioMedicine. (2023) 89:104485. doi: 10.1016/j.ebiom.2023.104485 36857860 PMC9970285

[11] Guo L Zhang Q Gu X Ren L Huang T Li Y . Durability and cross-reactive immune memory to SARS-CoV-2 in individuals 2 years after recovery from COVID-19: a longitudinal cohort study. Lancet Microbe. (2024) 5:e24–33. doi: 10.1016/s2666-5247(23)00255-0 38048805 PMC10789611

[12] Peng W Ma X Tan K Wang H Cong M Zhang Y . Evaluation of cross-neutralizing antibodies in children infected with omicron sub-variants. Lancet Regional Health - Western Pacific. (2023) 40:100939. doi: 10.1016/j.lanwpc.2023.100939 37953966 PMC10632766

[13] Ceban F Ling S Lui LMW Lee Y Gill H Teopiz KM . Fatigue and cognitive impairment in Post-COVID-19 Syndrome: A systematic review and meta-analysis. Brain Behavior Immun. (2022) 101:93–135. doi: 10.1016/j.bbi.2021.12.020 34973396 PMC8715665

[14] Rubino F Amiel SA Zimmet P Alberti G Bornstein S Eckel RH . New-onset diabetes in covid-19. N Engl J Med. (2020) 383:789–90. doi: 10.1056/nejmc2018688 32530585 PMC7304415

[15] Abbas U Kumar H Hussain N Ahmed I Laghari RN Tanveer M . Immune dysregulation in type 2 diabetes mellitus: Implications for tuberculosis, COVID-19, and HIV/AIDS. Infect Med. (2025) 4:100211. doi: 10.1016/j.imj.2025.100211 41216008 PMC12596640

[16] Lu Z-H Yu W-L Sun Y . Multiple immune function impairments in diabetic patients and their effects on COVID-19. World J Clin cases. (2021) 9:6969–78. doi: 10.12998/wjcc.v9.i24.6969 34540952 PMC8409204

[17] Viurcos-Sanabria R Escobedo G . Immunometabolic bases of type 2 diabetes in the severity of COVID-19. World J Diabetes. (2021) 12:1026–41. doi: 10.4239/wjd.v12.i7.1026 34326952 PMC8311488

[18] He Y Yin Y Zhang Y Yang H Jiang Z Hao F . Dynamics of neutralizing antibodies against COVID-19 omicron subvariants following breakthrough infection in southwest China between december 2022 and april 2024. Signal Transduction Targeted Ther. (2025) 10:242. doi: 10.1038/s41392-025-02319-3 40738879 PMC12310930

[19] Eshraghi R Bahrami A Karimi Houyeh M Nasr Azadani M . JN.1 and the ongoing battle: unpacking the characteristics of a new dominant COVID-19 variant. Pathog Global Health. (2024) 118:453–8. doi: 10.1080/20477724.2024.2369378 38884317 PMC11441051

[20] Holt RIG Cockram CS Ma RCW Luk AOY . Diabetes and infection: Review of the epidemiology, mechanisms and principles of treatment. Diabetologia. (2024) 67:1168–80. doi: 10.1007/s00125-024-06102-x 38374451 PMC11153295

[21] Li H Wang Y Ao L Ke M Chen Z Chen M . Association between immunosuppressants and poor antibody responses to SARS-CoV-2 vaccines in patients with autoimmune liver diseases. Front Immunol. (2022) 13:988004. doi: 10.3389/fimmu.2022.988004 36275639 PMC9579272

[22] Dai R Peng W Xu N Qin P Ding L Hua Q . Clinical characteristics and antibody response to omicron variants among solid carcinoma patients in China on the 2022.12–2023.4 wave of the COVID-19 pandemic. Front Immunol. (2024) 15:1476186. doi: 10.3389/fimmu.2024.1476186 39569188 PMC11576375

[23] Norouzi M Norouzi S Ruggiero A Khan MS Myers S Kavanagh K . Type-2 diabetes as a risk factor for severe COVID-19 infection. Microorganisms. (2021) 9:1211. doi: 10.3390/microorganisms9061211 34205044 PMC8229474

[24] Wang Y So HC Tsang NNY Kwok SK Cowling BJ Leung GM . Clinical profile analysis of SARS-CoV-2 community infections during periods with omicron BA.2, BA.4/5, and XBB dominance in hong kong: A prospective cohort study. Lancet Infect Dis. (2025) 25:276–89. doi: 10.1016/s1473-3099(24)00574-7 39419049

[25] Berbudi A Rahmadika N Tjahjadi AI Ruslami R . Type 2 Diabetes and its Impact on the Immune System. Curr Diabetes Rev. (2020) 16:442–9. doi: 10.2174/18756417mtaxgodqqy PMC7475801 31657690

[26] Chen X Hu W Ling J Mo P Zhang Y Jiang Q . Hypertension and diabetes delay the viral clearance in COVID-19 patients. Cold Spring Harbor, New York, USA: medRxv. (2020) 2020. doi: 10.1101/2020.03.22.20040774

[27] Takahashi T Ellingson MK Wong P Israelow B Lucas C Klein J . Sex differences in immune responses that underlie COVID-19 disease outcomes. Nature. (2020) 588:315–20. doi: 10.1038/s41586-020-2700-3 32846427 PMC7725931

[28] Collier DA Ferreira IATM Kotagiri P Datir RP Lim EY Touizer E . Age-related immune response heterogeneity to SARS-CoV-2 vaccine BNT162b2. Nature. (2021) 596:417–22. doi: 10.1038/s41586-021-03739-1 34192737 PMC8373615

[29] Wang Q Iketani S Li Z Liu L Guo Y Huang Y . Alarming antibody evasion properties of rising SARS-CoV-2 BQ and XBB subvariants. Cell. (2023) 186:279–286.e8. doi: 10.1016/j.cell.2022.12.018 36580913 PMC9747694

[30] Warpechowski J Leszczyńska P Juchnicka D Olichwier A Szczerbiński Ł Krętowski AJ . Assessment of the immune response in patients with insulin resistance, obesity, and diabetes to COVID-19 vaccination. Vaccines. (2023) 11:1203. doi: 10.3390/vaccines11071203 37515018 PMC10383449

