# Supplementary material for: Clinical characteristics of Chinese diabetic patients and their antibody responses to Omicron subvariants
Source: Front Immunol. 2026 Aug 12;17:1909061. doi: 10.3389/fimmu.2026.1909061 (PMC13507880; doi:10.3389/fimmu.2026.1909061)
Supplement: Supplementary file 1 [file Table1.docx]

| **Table S1. Factors associated with COVID-19 symptoms in the healthy controls** | | | | | | | | | | |
| --- | --- | --- | --- | --- | --- | --- | --- | --- | --- | --- |
| **Variable** | **COVID-19 infection** | | **General symptom** | | **Respiratory symptom** | | **Fever (≥38℃)** | | **Muscular soreness** | |
|  | N (%) | P | N (%) | P | N (%) | P | N (%) | P | N (%) | P |
| Sex | | | | | | | | | | |
| Male | 86(46.7%) | 0.352 | 66(42.9%) | 0.158 | 37(24.0%) | 0.657 | 57(37.0%) | 0.204 | 28(18.2%) | 0.664 |
| Female | 98(53.3%) |  | 66(42.9%) |  | 39(25.3%) |  | 56(36.4%) |  | 29(18.8%) |  |
| Age, years | | | | | | | | | | |
| 18-59 | 64(34.8%) | 0.585 | 48(31.2%) | 0.473 | 26(16.9%) | 0.891 | 43(28.0%) | 0.240 | 27(17.5%) | 0.016 |
| ≥60 | 120(65.2%) |  | 84(54.5%) |  | 50(32.5%) |  | 70(45.5%) |  | 30(19.5%) |  |
| BMI (kg/m2) | | | | | | | | | | |
| <25 | 130(71.0%) | 1.000 | 97(63.0%) | 0.357 | 57(37.0%) | 0.448 | 82(53.2%) | 0.733 | 41(26.6%) | 0.858 |
| ≥25 | 53(29.0%) |  | 36(23.4%) |  | 20(13.0%) |  | 32(20.8%) |  | 16(10.4%) |  |
| Vaccine(s) administered | | | | | | | | | | |
| Yes | 182(98.9%) | 1.000 | 130(84.4%) | 1.000 | 74(48.1%) | 0.169 | 111(72.1%) | 0.523 | 56(36.4%) | 0.525 |
| No | 2(1.1%) |  | 2(1.3%) |  | 2(1.3%) |  | 2(1.3%) |  | 1(0.6%) |  |

Data are presented as n (%). Statistical analyses were conducted using the chi-square test or Fisher’s exact test.

| **Table S2. Multivariable regression analysis of the association between diabetes status and antibody responses among all participants** | | | | |
| --- | --- | --- | --- | --- |
|  | Variable | B | 95%CI | P value |
| Anti-RBD IgG | Age, years | -0.0020 | -0.0072 — 0.0031 | 0.4396 |
|  | Sex | -0.0609 | -0.2331 — 0.1112 | 0.4870 |
|  | Vaccine(s) administered | 0.1506 | -0.2112 — 0.5125 | 0.4136 |
|  | Diabetes status | -0.2255 | -0.3998 — -0.0512 | 0.0114 |
| BA.4/5 | Age, years | -0.0249 | -0.0397 — -0.0102 | 0.0010 |
|  | Sex | -0.9431 | -1.4340 — -0.4526 | 0.0002 |
|  | Vaccine(s) administered | -0.5233 | -1.5540 — 0.5077 | 0.3190 |
|  | Diabetes status | -0.6225 | -1.1190 — -0.1258 | 0.0142 |
| XBB.1.5 | Age, years | 0.0025 | -0.0080 — 0.0130 | 0.6397 |
|  | Sex | -0.4296 | -0.7806 — -0.0786 | 0.0166 |
|  | Vaccine(s) administered | -0.5039 | -1.2420 — 0.2339 | 0.1801 |
|  | Diabetes status | -1.8310 | -2.1870 — -1.4760 | <0.0001 |

Data are presented as regression coefficients (B), 95% confidence intervals (95% CI), and P values. Multivariable linear regression analyses were performed among all enrolled participants to evaluate the association between diabetes status and antibody responses after adjustment for age, sex, and COVID-19 vaccination status. Diabetes status was coded as diabetic patients = 1 and healthy controls = 0, and sex was coded as male = 1 and female = 0. Neutralizing antibody titers were log₂-transformed before analysis. Statistical significance was defined as P < 0.05.

| **Table S3. Factors associated with SARS-CoV-2 antibody levels in the healthy controls** | | | | | | |
| --- | --- | --- | --- | --- | --- | --- |
| **Variable** | **Anti-RBD IgG** | | **BA.4/5** | | **XBB.1.5** | |
|  | Titer | *P* | GMT | *P* | GMT | *P* |
| Sex | | | | | | |
| Male | 1.9(0.8，2.4) | 0.512 | 551.3(414.3,733.6) | 0.005 | 40.1(30.4,53.0) | 0.062 |
| Female | 1.7(0.8，2.3) |  | 925.3(746.1,1148) |  | 61.4(47.2,79.8) |  |
| Age, years | | | | | | |
| 18-59 | 1.8(1.0，2.4) | 0.744 | 1165(876.5,1549) | <0.001 | 47.7(36.5,62.4) | 0.717 |
| ≥60 | 1.8(0.7，2.4) |  | 561.6(450.8,699.7) |  | 51.1(39.6,66.1) |  |
| BMI (kg/m^2^) | | | | | | |
| <25 | 1.9(0.8,2.4) | 0.185 | 747.5(603.1,926.3) | 0.627 | 51.9(41.4,64.9) | 0.730 |
| ≥25 | 1.5(0.8,2.4) |  | 676.9(483.0,948.5) |  | 48.1(33.5,69.2) |  |
| Vaccine(s) administered^a^ | | | | | | |
| Yes | 1.9(1.0,2.4) | <0.001 | 713.5(595.5,855.0) | 0.285 | 49.0(40.5,59.3) | 0.158 |
| No | 0.1(0.1,0.1) |  | - |  | - |  |
| COVID-19 infection | | | | | | |
| Yes | 1.8(0.8,2.4) | <0.001 | 872.7(759.4,1003.0) | <0.001 | 59.6(49.7,71.5) | <0.001 |
| No | 1.4(1.1,1.6) |  | 78.5(23.4,263.2) |  | 6.6(3.8,11.4) |  |

Data are presented as median (IQR). Statistical analyses were performed using the non-parametric Mann-Whitney U test. Data for pseudovirus neutralization titers (pVNT_50_) against Omicron subvariants (BA.4/5, XBB.1.5) and are presented as GMTs with 95% CI. Statistical significance for pVNT50 was determined using unpaired t-tests or one-way ANOVA following log10 transformation. A P value < 0.05 was considered statistically significant.

^a^The sample of participants without vaccine administered was very small sample sizes (n=2), the GMTs and 95% CIs was not feasible.

| **Table S4. Complete-case sensitivity analyses assessing the influence of BMI adjustment on antibody responses** | | | | |
| --- | --- | --- | --- | --- |
|  | **Variable** | **B** | **95%CI** | **P value** |
| **Anti-RBD IgG, model with BMI adjustment** | age | -0.0009 | -0.0068 — 0.0049 | 0.7498 |
|  | sex | 0.0560 | -0.1356 — 0.2476 | 0.5656 |
|  | Diabetes status | -0.2033 | -0.6066 — 0.2000 | 0.3217 |
|  | Vaccine(s) administered | -0.2464 | -0.9346 — 0.4418 | 0.4814 |
|  | COVID-19 infection | 1.6150 | 1.2200 — 2.0110 | <0.0001 |
|  | BMI | -0.1944 | -0.3992 — 0.0104 | 0.0627 |
| **Anti-RBD IgG, model without BMI adjustment** | age | -0.0012 | -0.0070 — 0.0047 | 0.6942 |
|  | sex | 0.0210 | -0.1679 — 0.2099 | 0.8268 |
|  | Diabetes status | -0.2219 | -0.6266 — 0.1828 | 0.2813 |
|  | Vaccine(s) administered | -0.2177 | -0.9084 — 0.4731 | 0.5355 |
|  | COVID-19 infection | 1.5990 | 1.2020 — 1.9960 | <0.0001 |
| **BA.4/5, model with BMI adjustment** | age | -0.0182 | -0.0341 — -0.0024 | 0.0240 |
|  | sex | -0.8590 | -1.3790 — -0.3388 | 0.0013 |
|  | Diabetes status | -1.8870 | -2.9820 — -0.7921 | 0.0008 |
|  | Vaccine(s) administered | -1.1540 | -3.0220 — 0.7145 | 0.2250 |
|  | COVID-19 infection | 3.1900 | 2.1150 — 4.2640 | <0.0001 |
|  | BMI | 0.0915 | -0.4645 — 0.6475 | 0.7462 |
| **BA.4/5, model without BMI adjustment** | age | -0.0181 | -0.0339 — -0.0024 | 0.0245 |
|  | sex | -0.8426 | -1.3520 — -0.3330 | 0.0013 |
|  | Diabetes status | -1.8780 | -2.9700 — -0.7865 | 0.0008 |
|  | Vaccine(s) administered | -1.1670 | -3.0310 — 0.6959 | 0.2184 |
|  | COVID-19 infection | 3.1970 | 2.1260 — 4.2690 | <0.0001 |
| **XBB.1.5, model with BMI adjustment** | age | 0.0096 | -0.0031 — 0.0223 | 0.1396 |
|  | sex | -0.3435 | -0.7614 — 0.0743 | 0.1067 |
|  | Diabetes status | -1.4300 | -2.3100 — -0.5509 | 0.0015 |
|  | Vaccine(s) administered | -1.5770 | -3.0780 — -0.0759 | 0.0396 |
|  | COVID-19 infection | 3.1640 | 2.3000 — 4.0270 | <0.0001 |
|  | BMI | 0.0002 | -0.4464 — 0.4469 | 0.9992 |
| **XBB.1.5, model without BMI adjustment** | age | 0.0096 | -0.0031 — 0.0222 | 0.1385 |
|  | sex | -0.3435 | -0.7527 — 0.0658 | 0.0996 |
|  | Diabetes status | -1.4300 | -2.3070 — -0.5536 | 0.0015 |
|  | Vaccine(s) administered | -1.5770 | -3.0730 — -0.0803 | 0.0390 |
|  | COVID-19 infection | 3.1640 | 2.3030 — 4.0250 | <0.0001 |

Data are presented as regression coefficients (B), 95% confidence intervals (95% CIs), and P values. Complete-case sensitivity analyses were performed among participants with available BMI data. For each antibody outcome, two multivariable linear regression models were constructed: one model adjusted for age, sex, diabetes status, COVID-19 vaccination status, and SARS-CoV-2 infection status, and the other model additionally adjusted for BMI category. Neutralizing antibody titers against BA.4/5 and XBB.1.5 were log₂-transformed before analysis. Statistical significance was defined as P < 0.05.
